# Supplementary figures and images for: Sporadic inclusion body myositis-derived myotube culture revealed muscle cell-autonomous expression profiles
Source: PLoS One. 2024 Aug 1;19(8):e0306021. doi: 10.1371/journal.pone.0306021 (PMC11293708; doi:10.1371/journal.pone.0306021)

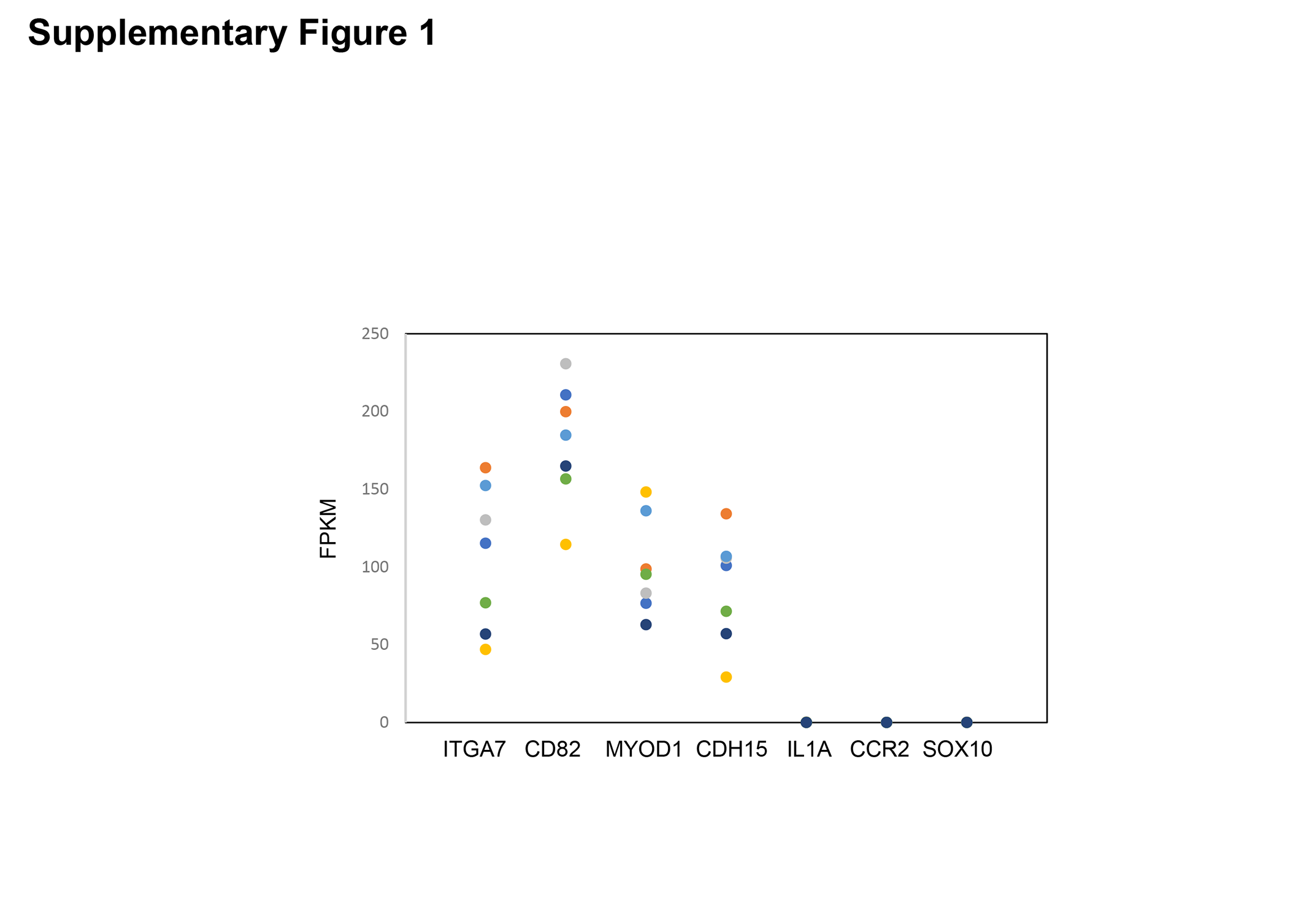

Supplement: S1 Fig — (TIF) [file pone.0306021.s001.tif]

**S1\_raw\_images**  
**Full blot of Figure 5A**

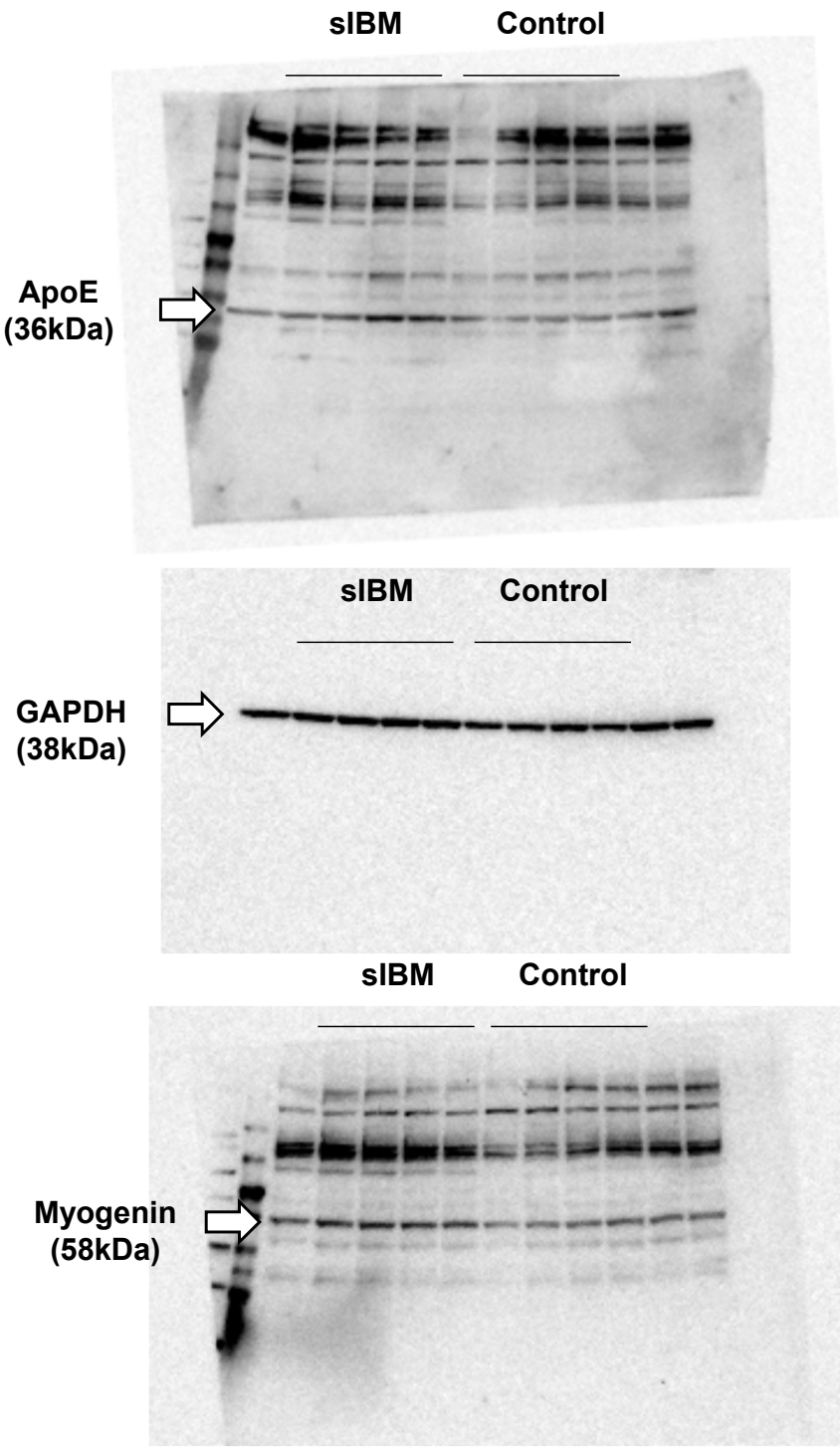

Supplement: S1 Raw images — (PDF) [file pone.0306021.s002.pdf]
